# Pathobiology of wound healing after glaucoma filtration surgery

**DOI:** 10.1186/s12886-015-0134-8

**Published:** 2015-12-17

**Authors:** Osamu Yamanaka, Ai Kitano-Izutani, Katsuo Tomoyose, Peter S. Reinach

**Affiliations:** Department of Ophthalmology, Wakayama Medical University, 811-1 Kimiidera, Wakayama, Wakayama 641-0012 Japan; Departments of Ophthalmology and Optometry Wenzhou Medical University, Wenzhou, 325027 People’s Republic of China

**Keywords:** Growth factor, Cytokine, TGF-β, Conjunctiva, Fibroblast, Fibrosis

## Abstract

Conjunctival and subconjunctival fibrogenesis and inflammation are sight compromising side effects that can occur subsequent to glaucoma filtration surgery. Despite initial declines in intraocular pressure resulting from increasing aqueous outflow, one of the activated responses includes marshalling of proinflammatory and pro-fibrogenic cytokine mediator entrance into the aqueous through a sclerostomy window and their release by local cells, as well as infiltrating activated immune cells. These changes induce dysregulated inflammation, edema and extracellular matrix remodeling, which occlude outflow facility. A number of therapeutic approaches are being taken to offset declines in outflow facility since the current procedure of inhibiting fibrosis with either mitomycin C (MMC) or 5-fluorouracil (5-FU) injection is nonselective. One of them entails developing a new strategy for reducing fibrosis induced by wound healing responses including myofibroblast transdifferentiation and extracellular matrix remodeling in tissue surrounding surgically created shunts. The success of this endeavor is predicated on having a good understanding of conjunctival wound healing pathobiology. In this review, we discuss the roles of inappropriately activated growth factor and cytokine receptor linked signaling cascades inducing conjunctival fibrosis/scarring during post-glaucoma surgery wound healing. Such insight may identify drug targets for blocking fibrogenic signaling and excessive fibrosis which reduces rises in outflow facility resulting from glaucoma filtration surgery.

## Introduction

It is estimated in 2010 that there were more than 60.5 million people worldwide with glaucoma and this number is expected to increase to reach 79.6 million by 2020 [1]. Glaucoma permanently damages optic neurons, leading to visual field declines and finally potentially causing blindness in patients that cannot be treated properly. Reducing the intraocular pressure is the only effective therapy to prevent visual impairment and blindness in hypertensive and normotensive individuals [2–4]. Commonly, the first therapeutic approach entails using topical agents that decrease aqueous humor production or promote outflow. A wide number of different options are available some of which are targeted to suppressing the activity of receptors regulating aqueous humor inflow and outflow facility. If the pressure lowering effects of these agents are not adequate, surgical intervention is suggested, i.e., laser treatment or filtration surgery [5]. Tube shunt surgery was first authorized and started to be performed in Japan in 2012 [6]. However, in Japan and in some other countries, trabeculectomy is still performed than a tube shunt. Nevertheless, in Europe and the US, the tube shunt is now the standard glaucoma filtration surgical procedure [7]. In any case, the fibrogenic and inflammatory processes are basically the same in both procedures. With filtration surgery, a scleral fistula is created to increase fluid drainage from the aqueous humor. This drained fluid accumulates underneath the conjunctiva creating a filtering bleb. Tissue fibrosis resulting from an overly driven wound healing response may impair filtering bleb formation and reduce aqueous humor outflow causing reversal of the initial decline in intraocular pressure. We deal here with the pathobiological subconjunctival wound healing responses induced by glaucoma filtration surgery, which affect the duration of the pressure lowering effect of this procedure. Another factor that we consider is the contribution made by different types of conjunctival responses to injury that offset declines in IOP achieved by this procedure.

## Mechanism of fibrosis/scar of anterior ocular segments

### Overview

The pathophysiological mechanisms activated by injury inducing tissue fibrosis are the same in all non-nervous tissues and organs of the human body. For example, injury-induced corneal and conjunctival fibrotic development mirrors the sequel occurring in skin. In these tissues epithelial and mesenchymal cells undergo during, wound healing complex and dynamic changes. Their exposure to an inflammatory milieu promotes phenotypic changes leading to increases in proliferation and migration, and tissue remodeling. Inflammation occurs during an early phase of wound healing and is attributable to immune cell activation of neutrophils and macrophages causing them to elaborate proinflammatory cytokines and chemokine and infiltrate into a wound. Ocular surface stromal cells are normally quiescent, but they become activated at a wound by various proinflammatory cytokines released by infiltrating inflammatory cells. For example TGF-β release induces mesenchymal cell and fibroblast activation leading them to subsequently reenter the cell cycle, migrate and undergo transformation into myofibroblasts. These transformed cells elaborate a host of mediators which degrade the extracellular matrix (ECM) and components that frequently fail to restore its original organization. ECM remodeling is attributable to excessive accumulation of matrix components consisting of an interlocking meshwork of collagen with other ECM components such as proteoglycans and glycosaminoglycans (GAGs), which are one of its side chain constituents. Characteristic of this remodeling process is tissue granulation accompanied by inflammatory cell influx, neovascularization and altered vascular permeability

Myofibroblasts elaborate contractile proteins whose contractile force also contributes ECM reorganization and loss of tissue functionality. They are derived through TGF-β-activated mesenchymal cells, i.e., subconjunctival fibroblasts transdifferentiation at the site of injured tissue. It is unclear whether or not bone marrow-derived fibrocytes make a significant contribution to tissue remodeling. Epithelial or endothelial cells can also under dedifferentiation and transformation into myofibroblast phenotype after injury [8], indicating there are several potential sources of myofibroblasts. At a fibrotic/scar lesion, persistent inflammation and myofibroblast formation must be subdued to permit normal tissue function restoration [9, 10]

### Histopathology of filtering bleb following wound healing post-trabeculectomy

Conjunctiva fibrosis/scar formation during wound healing induced by ocular surgery reduces aqueous filtering in the surgically treated glaucomatous eye (discussed below) and shrinks the conjunctival sac during healing following various ocular surgeries. For example, in the latter case the loss of conjunctival flexibility due to fibrosis hinders wound surface resurfacing rendering the tissue vulnerable to microbial infection.

Currently, intraocular pressure lowering is the only evidence-based effective glaucoma treatment [2, 3]. In glaucoma patients unsuccessfully treated with glaucoma medications, filtration surgery is performed for the purpose of reducing intraocular pressure. Trabeculectomy is the gold standard for glaucoma filtration surgery. Although this procedure was first introduced in the 1960’s by Cairn, it is still common surgical procedure in Japan and in some other countries [5]. Filtration surgery via scleral surgical fistula implantation is a drainage procedure, which diverts intraocular aqueous humor fluid to underneath the conjunctiva. Bleb formation in the conjunctiva is indicative of a successful procedure. If the fistula remains in place, a favorable prognosis is anticipated assuming the fistula remains patent and a working filtering bleb is retained. In other words, the surgical outcome depends on filtration sufficiency through a sclerostomy that is insensitive to differences in the conjunctival wound healing response induced by surgery. However, conjunctiva fibrosis/scar formation often disrupts aqueous humor drainage into the bleb, which is an undesirable complication following filtering surgery.

Another problem making management of glaucoma somewhat problematic is that a commonly prescribed drug class, prostaglandin analogues, can induce inflammation by upregulating proinflammatory cytokine gene expression even though they stably reduce intraocular pressure [11, 12]. There are several reports describing an inverse correlation between subclinical inflammation and filtration surgery outcome. However it is still uncertain if such a relationship exists between conjunctival inflammation and prognosis of surgery [13, 14]. Subclinical inflammation may be undesirable for wound healing, but further studies are needed to determine if there is an association between other types of glaucoma medication, dosage duration and ocular inflammation.

### Roles of aqueous humor-derived growth factors/cytokines on conjunctival bleb scarring

Variations in the aqueous humor TGF-β2 ratio between its active and inactive forms are postulated to modulate the filtering bleb, and fibrotic reactions induced by local fibroblasts [15], Increases in the ratio of this growth factor occurs in tissues compromised by injury. Hu et al. reported that aqueous humor VEGF levels increase in neovascular and primary open angle glaucoma patients [16]. Park et al. reported that VEGF induces TGF-β1 to rise in the subconjunctival scar tissue after trabeculectomy and suggested that VEGF increases can stimulate the TGF-β1/Smad/Snail signaling pathway leading to myofibroblast transformation [17]. TGF-β1 and β2 are both capable of promoting fibroblast-myofibroblast transdifferentiation and ECM remodeling (discuss in below). Sawada et al. reported that glaucoma patients had higher levels of tumor necrosis factor-α (TNF-α), than in normal controls [18]. TNF-α is one of the major pro-inflammatory growth factors besides proinflammatory interleukins (ILs). For example, IL-6 and IL-8 levels are significantly elevated in glaucoma patients [19, 20].

It is to be noted that increases in a lens epithelial cell-derived factor also affects a wound healing reaction in filtering bleb tissue. Such an effect was observed after a trabeculectomy was performed in one eye after having received an intraocular lens implant. Phacoemulsification extraction of a cataractous lens epithelial cells up regulate macrophage-chemoattractant protein-1 (MCP-1) levels in the aqueous humor [21]. MCP-1 induces macrophage infiltration into local tissue, leading there to rises in proinflammatory and fibrotic growth factors. Monocyte-macrophage infiltration also makes an important contribution to tissue fibrosis [22]. Activated macrophages in an injured tissue produce pro-inflammatory/pro-fibrogenic growth factors and cytokines, i.e., VEGF, PDGF, TGF-β and TNF-α [23–25]. Excessive ECM elaboration and rises in cytokines/growth factors lead to fibrosis. Therefore, drug management of macrophage activity could become a promising target to reduce injury-induced fibrosis. Serum amyloid P (SAP) or pentraxin 2 (PTX2) is a member of the pentraxin family and is thought to be a potent modulator of the monocyte macrophage response [26]. Recently a recombinant reagent of human SAP, PRM-151 was developed and its effect was evaluated on fibrotic diseases such as idiopathic pulmonary fibrosis and myelofibrosis. It may be a promising agent for preventing scar formation after glaucoma surgery [27, 28]

### Roles of growth factors/cytokines expressed in local fibroblasts and inflammatory cells on conjunctival bleb scarring

Besides increasing aqueous humor growth factor and cytokine levels, injury to (sub)conjunctival tissue resulting from surgical intervention activates local tissue cells, e.g., fibroblasts and also induces proinflammatory neutrophils and macrophages infiltration into a wound. They express a large number of growth factors and proinflammatory cytokines promoting tissue inflammation and fibrosis. In the very early phase post-injury or surgery, local fibroblasts are rapidly activated by signals that in turn sensitize various sensors of external stimuli to undergo activation. These sensors include cation channels receptors that elicit activation of downstream linked signaling pathways. Tissue damage also directly transforms latent TGF-β stored in ECM into an active form. Chemokines and growth factors/cytokines secreted by local fibroblasts and activated TGF-β stimulate fibroblast and monocytes-macrophages activation. All of these altered cell types elicit factors modulate the sequential linked events controlling wound healing [29].

### Potential targets for modulation of wound healing in conjunctiva post-trabeculectomy

Currently, adjunctive application of mitomycin C (MMC) or 5‑fluorouracil (5‑FU) after filtering surgery is performed to attenuate postoperative subconjunctival fibroblasts proliferation for suppressing excessive bleb scarring. These adjunctive anti-metabolites have much improved the success rate of trabeculectomy [30]. These drugs are used post or during surgery to prevent subconjunctival fibrosis. 5-FU is a pyrimidine analog that was originally applied as a cancer treatment. 5‑FU acts as an inhibitor of the enzyme thymidylate synthetase during the S phase of cell cycle. This agent can be used during or post surgery. Several studies describe 5‑FU efficacy use after glaucoma surgery [31–33]. MMC inhibits DNA synthesis and it was originally used as a systemic anti-tumor agent isolated from Streptomyces caespitosus. Various studies demonstrated MMC increases trabeculectomy success [34–36]. However, the effects of MMC on cell proliferation are not cell type specific and in excessive amounts it can be cytotoxic. Furthermore, these agents can induce acellular and avascular bleb formation, which are susceptible to leakage and microbial infection [37, 38]. Therefore more targeted and less toxic agents are still needed. We next review promising drug targets whose modulation affect wound healing responses to injury.

### Interleukins (ILs)

Interleukins (IL) are a group of cytokines with complex immunomodulatory functions including cell proliferation, maturation, migration and adhesion control as well as having important roles in regulating immune cell differentiation and activation [39]. Some of the IL family members are well recognized as pro-inflammatory cytokines concerned with fibrotic or inflammatory diseases, i.e., IL-1, 6 and IL-8. These ILs are designated therapeutic drug targets and several inhibitors are under investigation to determine if they are effective in the prevention/treatment of unfavorable tissue fibrosis/scarring. Piquet et al. administered an IL-1 receptor antagonist (IL-1ra) to mouse pulmonary fibrosis model mice and showed that it prevented collagen deposition and improved lung fibrosis [40]. Tocilizumab, an IL-6 receptor inhibitor, in a rheumatoid arthritis clinical study OPTION study), decreased inflammation and tissue scarring [41]. This finding prompted us to hypothesize that this inhibitor is an effective agent for reducing inflammation and scarring following glaucoma surgery.

Some of the IL members, i.e., IL-7, IL-10 or IL-22, also exhibit anti-fibrotic effects on cells. IL-7 binding to its receptor activates signaling that counteracts TGFβ/Smad signal and thus suppresses ECM expression by fibroblasts [42]. IL-10 reportedly suppresses fibrotic reaction in experimental liver fibrosis model in rats. Gene ablation of IL-10 exaggerates the pathology of experimental colitis or renal interstitial fibrosis induced by unilateral ureter ligation in mice, also indicating that this IL exhibits anti-fibrotic or anti-inflammatory effects [43, 44].

IL-22 is another anti-fibrotic/anti-inflammatory IL and is an IL-10 family member. For example, systemic administration of recombinant IL-22 has an anti fibrotic effect in a rat liver fibrosis model presumably caused by Stat3 signaling inhibition [45]. In an injured conjunctiva, these pro- or anti-fibrotic ILs presumably have complex effects on local fibroblasts and inflammatory cell behavior.

### Vascular endothelial growth factor (VEGF)

Vascular endothelial growth factor (VEGF) is a potent mediator of vascular homeostasis, i.e., angiogenesis, vasculogenesis and vascular endothelial cell permeability [46, 47]. Angiogenesis is an important component of wound healing leading to fibrosis. Currently several anti VEGF agents are being evaluated for use in treating age-related macular degeneration and other retinal diseases [48]. As VEGF concentration is elevated after glaucoma surgery and plays a key role promoting cell proliferation, it could be also a drug target for preventing excess fibrosis/scarring in post-surgery (sub)conjucntival tissue [49]. Kahook et al. demonstrated in humans that subconjunctival injection after glaucoma surgery of an anti VEGF antibody, bevacizumab, maintained the bleb [50]. Coote et al. showed that this procedure following cataract surgery prevented bleb failure and reduced its vascularization [51]. Several other reports suggest the possibility that anti-VEGF agents in combination with MMC and 5-FU have a synergistic effect in improving glaucoma surgery outcome [52, 53]. Moreover, topical bevacizumab application prevented MMC side effects since it reduces the exposure time to this mitotic inhibitor, which is injected into scleral and subconjunctival tissues at the site of trabeculectomy. In addition, it was found that this procedure improved bleb survival [54]. Van Bergen and et al. showed that VEGF isoforms play a different role in scar formation since injecting Pegaptanib, an RNA aptamer directed against VEGF_165_, a VEGF subfamily member involved in pathological angiogenesis, improved surgical outcome using a rabbit model of trabeculectomy [55]. Overall VEGF has a pivotal role in determining the wound healing response to glaucoma surgery. The optimum route of administration and dosing regimen of anti-VEGF therapy is still requires careful consideration [56].

### Platelet-derived growth factor (PDGF)

The PDGF family of growth factor isoforms consists of five different disulphide-linked dimers built up of four different polypeptide chains encoded by four different genes. Besides inducing macrophages and fibroblasts to proliferate and migrate into a wound site, their upregulation leads to fibrosis [57–59]. Akiyama et al. applied ARC126 and ARC127, PDGFβ inhibitors, in a proliferative vitreoretinopathy (PVR) model and reduced both epi-retinal membrane formation and retinal detachment [60]. The same strategy may be useful for managing glaucoma surgery.

### Connective tissue growth factor (CTGF)

TGF-β upregulates Connective Tissue Growth Factor (CTGF) expression, which is one of the most important fibrogenic cytokines. As TGF-β mediates through CTGF persistent fibrosis, drug targeting either CTGF or TGF-β signaling may also prevent declines in filtering bleb size by reducing scar tissue formation [61–63]. Blocking CTGF has already successfully reduced conjunctival scarring disorders, e.g., vernal conjunctivitis or Stevens-Johnson syndrome [64].

### Matrix metalloproteinases (MMPs)

MMPs are a group of proteolytic enzymes degrading most extracellular matrix proteins during ECM remodeling. Their activity is dysreglulated in connective tissue matrices diseases such as arthritis, tumorigenesis and ulceration making them a therapeutic target for reducing scar formation [65–67]. Wong et al. demonstrated that a MMP inhibitor, GM6001, significantly reduced scar formation post glaucoma surgery in rabbits [68]. Inhibition of MMP could provide another therapeutic option for reducing fibrosis in post glaucoma filtration surgery.

### Lysyl oxidase (LOX) and lysyl oxidase like proteins (LOXL)

Lysyl oxidase (LOX) and lysyl oxidase-like (LOXL) are ECM enzymes crosslinking substrates such as collagen and elastin, which leads to fibrosis [69, 70]. There are several lysyl oxidase family members, LOXL1, 2, 3 and 4 and each one of them has a biological function. An anti LOXL2 monoclonal antibody (AB0023) reduced inflammation as well as growth factor production and suppressed TGF-β signaling in xenograft tumor models [71]. Van Bergen et al. applied an anti LOXL2 monoclonal antibody (GS-607601) to rabbits undergoing glaucoma surgery and it reduced both inflammation as well as fibrosis. LOXL2 could be a promising therapeutic target for reducing scar formation after glaucoma surgery [72].

### Rho kinase signal

Rho-associated protein kinases (ROCK 1 and 2) are downstream components of Rho-GTPase Rho mediated signaling and play an important role in cytoskeletal organization. They also control cellular morphology migration and motility [73]. Honjo et al. showed that topical application of the ROCK inhibitor, Y-27632, improved the outcome of experimental glaucoma filtration surgery presumably by suppressing fibrogenic collagen deposition in tissue around blebs [74]. Rac1 is a low molecular weight Rho GTPase that is essential for cell motility and wound healing [75]. Tovell et al. demonstrated that either inhibiting Rac1 with NSC23766 or siRNA mediated gene silencing reduced conjunctival tissue fibrosis and collagen matrix contraction [76]. In Japan, a Rho kinase inhibitor is already approved for use to lower IOP in glaucoma patients. It remains to be determined if this inhibitor also has anti-fibrosis effects on the wound healing process following filtration surgery.

### Secreted protein acidic and rich in cysteine (SPARC)

SPARC is a 43 kDa collagen-binding matricellular glycoprotein contributing to ECM organization as well as cell migration mediation. These functions are manifested by modulating cellular interactions with the surrounding ECM [77, 78]. Its involvement in such control is evident since following SPARC knockdown cell movement and collagen gel contraction declined. Furthermore, TGFβ2-driven up regulation of type I collagen and fibronectin expression in vitro was suppressed [79]. In mice liver fibrosis models, SPARC-null mice inflammation and fibrosis were reduced although cellular mechanisms underlying these effects remain to be more fully elucidated [80]. In the eye, SPARC loss of function preserved and prolonged conjunctival bleb survival following experimental filtration surgery [81]. These effects suggest that reducing SPARC expression could be a novel option for suppressing (sub)conjunctival fibrosis.

### Angiotensin II

Recently it became apparent that angiotensin II is one of the common factors causing liver, kidney, heart and ocular fibrosis [82–85]. Angiotensin II has many biological functions besides including increasing cell proliferation, apoptosis, migration, inflammatory responses and ECM remodeling [86, 87]. Angiotensin-converting enzyme inhibitors and angiotensin receptor (AT1) antagonists suppressed vascular damage by inhibiting tissue fibrosis [88]. As for the anterior ocular segment, angiotensin II induced corneal myofibroblasts survival via activating NF-κB signaling, which promoted corneal fibrosis [84]. This might also be the case in (sub)conjunctival tissue. Shi et al. reported that angiotensin II levels were elevated along with its cognate receptors after glaucoma surgery in rabbits and angiotensin II promoted cell proliferation and migration in vitro. They suggest that angiotensin II may play an important role in wound healing after trabeculectomy and it can be a promising drug target to prevent scar formation during post filtration surgery [89].

### Transient receptor potential (TRP) channels

Transient receptor potential (TRP) channels are polymodal receptors that are commonly expressed in human tissues including the eye [90]. There are 28 different TRP channels that are subdivided into seven different subfamilies (TRPA, TRPC, TRPM, TRPML, TRPN, TRPP, and TRPV) [91]. They are activated by multiple endogenous and external stimuli and mediate pertinent wound healing functions. These receptor-induced responses include cell proliferation and migration stimulation along with inducing immune cell activation, tissue infiltration and fibrosis [92, 93]. Activation of some of the TRP channel subtypes contributes to liver, lung, kidney and heart fibrotic disease induction [94]. Okada et al. reported that in an alkali-burn mouse wound healing model TRPV1 antagonist treatment suppressed fibrosis during healing cornea and inhibited myofibroblast transdifferentiation in vitro using ocular fibroblast [95]. This improved wound healing response induced by an alkali burn was replicated in homozygous TRPV1 knockout mice. TRPV1 activation by injury is dependent on TGFβ receptor stimulation inducing transactivation of this ion channel through a p38MAPK Smad2 linked signaling pathway loop. Increasing the open time of this channel enhances intracellular Ca^2+^ influx, which triggers myofibroblast transdifferentiation and rises in proinflammatory cytokine release along with fibrosis [96]. Loss of TRPA1 function in mice also reduced inflammation and fibrosis during wound healing following an alkali burn in mice [97]. Developing novel selective TRP channel antagonists for suppressing injury-induced TRPV activation may be another novel option for improving the outcome of fibrogenic wound healing.

### Transforming growth factor-β (TGF-β) 

Among the wound healing promoting cytokines/growth factors, TGF-β is the most efficacious mediator of conjunctival scarring elicited by injury. There are many ongoing experimental and clinical trials evaluating drug options to suppress TGF-β-induced scar formation. Tranilast suppresses collagen production by specifically interfering with TGF-β activation of this response [98]. Chihara et al. used Tranilast in a pilot study after glaucoma surgery and found that it improved filtering bleb conditions [99]. TGF-βtype II receptor siRNA gene silencing suppressed fibronectin production and cell migration in vitro. In a mouse model, this procedure reduced the inflammatory response and matrix deposition post glaucoma surgery [100]. On the other hand, in clinical trials applying an anti TGF-β2 neutralizing antibody against failed to suppress fibrosis/scar after filtering surgery [101]. One possible explanation for this negative result is that the aqueous humor contains abundant TGF-β2, whereas TGF-β1 and β2 are produced by cells residing in the filtering bleb tissue [102]. Thus, it may be necessary to target all TGF-β family members rather than each individual TGF-β isoform for achieving a more desirable therapeutic outcome.

TGF-β isoforms activate cell surface TGF-β serine/threonine kinase receptors linked to signal transduction networks involving mitogen-activated protein kinase (MAPK)/ Extracellular Signal-Regulated Kinase (ERK), p38MAPK, C-Jun-N-terminal kinase (JNK), and Smads. Therefore, blocking TGF-β stimuli at the receptor level may potentially compromise epithelial healing on the ocular surface. Alternatively, it might be preferable to selectively block downstream signals induced by TRPV1 activation such as p38MAPK and Smad2/3/4/ complexation within mesenchymal cells and fibroblasts and thereby preserve the TGF-β signaling mediating maintenance of epithelium homeostasis.

For now, we focused on blocking the Smad pathway because of its involvement in triggering fibrosis induced by TGF-β. Regardless of the ligand isoform, blocking Smad2/3 signal suppressed TGF-β/Smad signaling pathways, allowing us to bypass the tissue-specific distribution of each TGF-β isoform in situ. Smad7 is one of the most important key players suppressing the TGF-β signaling mediated by Smads. Promising strategies have been developed to induce Smad7 expression by small molecule agents, e.g., IL-7, emodin and interferon-gamma and gene therapy prevent conjunctival fibrosis/scarring [42, 103–106]. Indeed, Smad7 adenoviral vector gene transfection into human subconjunctival fibroblasts inhibited TGF-β1induced up-regulation of fibrogenic and inflammatory cytokines [105]. Moreover, Smad7 gene transfer also attenuated the fibrogenic reaction in a healing, incision-injured, mouse conjunctiva, suggesting that this strategy might have a therapeutic potential in the prevention of excess scarring in the post-trabeculectomy filtering bleb [105]. Additional studies are required to delineate the roles of growth factors in controlling changes in ECM organization and behavior subsequent to injury induced by surgery. As TGF-β is a major player in inducing the untoward physiological and pathological changes associated with ocular wound healing, new strategies are needed to selectively reduce ocular fibrosis and inflammation.

### Conclusion and future perspectives

Even though ophthalmologists have developed many modifications improving glaucoma surgery, the outcome of this procedure still is variable due to limited success in selectively suppressing (sub)conjunctival fibrosis. The anti-proliferative drugs, MMC and 5-FU, are somewhat effective in reducing fibrosis, but their side effects can be worrisome due to their lack of cell type specificity in inducing cytotoxic effects besides inhibiting cell proliferation. These limitations indicate that novel drugs must still be developed to improve long term glaucoma medical management subsequent to this procedure. Their development hinges on first identifying targets whose modulation will selectively reduce injury-induced fibrosis and inflammation. This need is being met in part by delineating the various intracellular signal transduction pathways activated by a set of growth factors and cytokines involved in mediating wound healing. Moreover, through characterizing the pattern of changes in specific growth factors and cytokine expression induced by injury specific candidates have been identified for drug targeting. Another hurtle is that drug delivery systems need to be developed for overcoming the anterior ocular surface barrier function permitting sustained localized drug release. Excellent review articles are available on the pathobiology and clinical problems in bleb fibrosis. These articles deal with the roles of other growth factors or cytokines not addressed in this review [107, 108]. We hope that this presentation provides meaningful insight helping efforts to develop new promising anti-fibrotic and inflammatory agents that are more specific, safer and personalized facilitating glaucoma surgery-induced wound healing outcome by reducing attendant fibrosis and inflammation.
